# Disinformation and Epidemics: Anticipating the Next Phase of Biowarfare

**DOI:** 10.1089/hs.2020.0038

**Published:** 2021-02-18

**Authors:** Rose Bernard, Gemma Bowsher, Richard Sullivan, Fawzia Gibson-Fall

**Affiliations:** Rose Bernard, MA, and Gemma Bowsher, MBBS, are Research Associates; and Richard Sullivan, PhD, is Co-Director; all in Conflict and Health Research Group, Department of War Studies, King's College London, London, UK. Fawzia Gibson-Fall, MSc, is a Research Student, School of Politics and International Relations, Queen Mary University of London, London, UK.

**Keywords:** Disinformation, Biowarfare, Epidemics, Infodemics, Public health preparedness/response, Infectious diseases

## Abstract

While biological warfare has classically been considered a threat requiring the presence of a distinct biological agent, we argue that in light of the rise of state-sponsored online disinformation campaigns we are approaching a fifth phase of biowarfare with a “cyber-bio” framing. By examining the rise of measles cases following disinformation campaigns connected to the US 2016 presidential elections, the rise of disinformation in the current novel coronavirus disease 2019 pandemic, and the impact of misinformation on public health interventions during the 2014-2016 West Africa and 2019-2020 Democratic Republic of the Congo Ebola outbreaks, we ask whether the potential impact of these campaigns—which includes the undermining of sociopolitical systems, the delegitimization of public health and scientific bodies, and the diversion of the public health response—can be characterized as analogous to the impacts of more traditional conceptions of biowarfare. In this paper, we look at these different impacts and the norms related to the use of biological weapons and cyber campaigns. By doing so, we anticipate the advent of a combined cyber and biological warfare. The latter is not dependent on the existence of a manufactured biological weapon; it manages to undermine sociopolitical systems and public health through the weaponization of naturally occurring outbreaks.

## Introduction

Biological warfare has classically been viewed as an emergent threat arising from 4 distinct eras: pregerm theory, applied microbiology, industrial microbiology, and molecular biology and biotechnology.^1^ In light of today's disinformation campaigns that target public health measures and institutions, and, particularly, given the rise of global antivaccination campaigns and the undermining of contemporary domestic and international responses to epidemics and pandemics, we suggest that we are entering into a fifth era of biowarfare, one that incorporates the use of cyber capabilities and does not depend on the existence of a manufactured biological weapon *per se*. Biowarfare in the fifth era aims to undermine sociopolitical systems through social, political, and economic means by “weaponizing” or “virtually escalating” natural outbreaks, rather than directly inducing mortality and morbidity in populations through the deployment of harmful biological agents.

In late December 2019 and early January 2020, reports started emerging of a new coronavirus outbreak in Wuhan, China. This outbreak of novel coronavirus disease 2019 (COVID-19), which it was eventually named, was declared a public health emergency of international concern on January 30. A hallmark of this rapidly evolving pandemic has been the constant production of information from political, scientific, and lay arenas, describing often contradictory findings relating to the natural history, epidemiology, and clinical outcomes of COVID-19. Governments have employed these information products in highly disparate manners to enact mitigation and containment measures as well as media and communications strategies designed to contain the primary and secondary impacts of the pandemic.

In this context, high levels of scientific reporting and official guidance are contrasted against a vast swathe of media reporting, conflicting statistical interpretations, rumors, and theories. It is important to distinguish between disinformation and misinformation: misinformation is typically classified as “accidental falsehood,” or wrong and misleading information shared without malice, while disinformation is “deliberate falsehood,” or wrong or misleading information shared in full knowledge of its falsehood, often with malicious intent.^2^ The prevalence of misinformation and disinformation has been so significant during this pandemic that at the Munich Security conference on February 15, 2020, World Health Organization (WHO) Director-General Dr. Tedros Adhanom Ghebreyesus declared: “We're not just fighting an epidemic; we're fighting an infodemic.”^3^ Furthermore, in an interview with the *Lancet*, WHO Director of Infectious Hazards Management Sylvie Briand described outbreaks being accompanied by “a tsunami of information, but also within this information we have misinformation, rumours, etc.”^4^

While misinformation and outbreaks have long coexisted, this phenomenon has been disproportionately amplified in the last decade by a combination of social media, the normalization of fake news, and the delegitimization of scientific expertise. In particular, during the COVID-19 outbreak, US intelligence agencies and EU officials have attributed disinformation, including sustained social media posts claiming that the outbreak was caused by the United States, to Russian and Chinese disinformation campaigns.^5^ These active disinformation campaigns, combined with misinformation spread by social media, are likely to divert the course of the outbreak by amplifying mistrust of official reporting and the rejection of scientific evidence by the general public. The course of this “infodemics,” propagating alongside the COVID-19 pandemic, must be seen as a dual pathway of harm: concerted disinformation campaigns using cyber warfare techniques herald a new fifth era of biowarfare phenomena.

As reports of global disinformation campaigns driven by individual nation-states escalate, the consequences of these campaigns should be examined within the specific context, taking into consideration the securitization of health and biosecurity. This new era of biowarfare is emergent and has not yet been used to full extent; however, we argue that certain necessary conditions for its development have now been reached. These conditions are: (1) the weaponization of online fake news campaigns, with wide reach; (2) the potential impact of these campaigns to have significant negative impact on public health; (3) the exacerbating effect of social media misinformation and disinformation during an epidemic; and (4) the delegitimization of science and mistrust of officials. We argue that the presence of these contextual factors has made it possible for nation-states to wage biowarfare by achieving effects analogous to those of traditional biowarfare without deploying a traditional biological agent. We sustain that the potential impacts of these campaigns should, therefore, be analyzed within the context of a cyber biowarfare framework to apprehend the usefulness of the thresholds and limiting conditions of conventional biowarfare in this emerging context.

## Biowarfare

Traditionally, the use of biological weapons has been termed as either “biological warfare,” which is conducted by nation-states as campaigns to weaken and undermine an opponent, or “bioterrorism,” which aims to cause disruption and panic within a population.^6,7^ Although the category of biocrime also exists, it is largely defined as the actions of nonstate actors for profit.^8^ The diverse end aims, methods notwithstanding, of biowarfare and bioterrorism, therefore, can be conceived as:

1. the causing of fear and terror, and economic and political disruption, and civil unrest in a target population for political, ideological, or religious goals;^9^ and
2. the incapacitation of an enemy force or population either in a field of war or a domestic population.^10^

In traditional definitions of biowarfare and bioterrorism, these aims have been achieved and defined by the use of a biological agent or the threat of use of a biological agent; however, as we move into the third decade of the 21st century, these aims can be achieved by a sophisticated online disinformation and misinformation campaign: in particular, the threat of use of a biological agent, without the deployment of a biological agent *per se*. This has been made achievable by the convergence of conditions that have created an environment where it is not only possible to achieve these aims by cyber bionexus, but it is politically preferable for hostile actors to direct their efforts in this domain.

## Weaponization and Reach of “Fake News” Campaigns

The use of the term “fake news” refers more specifically to the online phenomena of spreading misinformation and disinformation masquerading as news reports or factual reports, enabled by the newsfeed functions on popular media such as Twitter and Facebook. It is important to note that the use of disinformation has been an enduring nation-state tactic to persuade “friends” as much as to fight enemies. In 1941, British Security Coordination, run by the British Secret Intelligence Services, ran a multipronged disinformation campaign to change the attitudes of the then isolationist United States and bring them into the war. This involved planting pro-British and anti-German stories in US newspapers and on US radio stations, and planting a map allegedly showing German plans to occupy South America to be discovered by the Allies.^11^ During World War II, the United States established “rumor clinics” to combat rumors allegedly propagated by Axis powers to threaten civilian morale.^11^ Information operations have been a practiced tactic by Russia since the Soviet Union and the Cold War—in 1984, KGB agents allegedly posed as Ku Klux Klan members to distribute inflammatory material and exacerbate racial tensions in Los Angeles.^12^

Disinformation is a well-established tactical and strategic approach. In security literature, however, fake news has mainly been associated with the online manifestation of this phenomenon, which utilizes the full extent of social media as a political tool to achieve nation-state goals. This approach has been used by Russia in an ongoing operation against the United States and western institutions since 2012.^13^ This operation sought to not only undermine US elections but also the wider faith in North Atlantic Treaty Organization and the “western” democratic project through the use of asymmetrical, nonmilitary means.^14^ Fake news as a cyber phenomenon became more well known following the US presidential elections in 2016, during which Russia was associated with a coordinated social media campaign using fake user accounts and networks of bots to divert the election process.^15^ Despite repeated subsequent denials of Russian involvement by the US presidential administration, it is likely that fake Facebook campaigns designed to polarize the electorate reached approximately 126 million Americans.^16^ Google reportedly identified US$4,700 worth of advertising on their platform, along with 18 fake YouTube channels, and Twitter found and took down 2,753 accounts.^16^ Facebook called this fake news campaign “the weaponization of misleading information and falsehoods in aid of geopolitical goals.”^17^

Posts made by the network of accounts or social media bots maintained by the Kremlin during this campaign were not necessarily politician or party specific, instead, they exploited a variety of polarizing issues within the United States to amplify divisions and increase the partisanship of politics, thereby weakening trust in the US establishment and potentially discrediting wider western democratic systems. For example, during the electoral campaign, a Russia-attributed Facebook account called “Heart of Texas” organized a protest called “Stop the Islamization of Texas”; a second Russia-attributed account called “United Muslims of America” organized a demonstration at exactly the same time and the same place.^14^ The identified associated strategy was “to take a crack in [US] society and turn it into a chasm.”^18^

Since the popularization of this online tactic and its evident impact on the US elections, other nations have similarly weaponized online fake news. A report by the Computational Propaganda Research Project in 2019 identified that social media manipulation campaigns had taken place in 70 countries globally. Additionally, 7 countries—China, India, Iran, Pakistan, Russia, Saudi Arabia, and Venezuela—had been observed running state-sponsored information operations on Facebook and Twitter.^19^ An Iranian-state connected fake news operation dubbed “Endless Mayfly” has been identified as operational since at least as early as 2016, and has been used to systematically spread propaganda and rumors about Israel, Saudi Arabia, and the United States.^20^ In 2019, misinformation was considered a significant threat to India's elections and disinformation campaigns against European elections and the British referendum on exit from the European Union were identified as substantial security risks.^21,22^ The link between disinformation and misinformation in these campaigns is crucial. While nation-state campaigns are usually associated with disinformation (the use of deliberate falsehood) they often play on existing tropes, stereotypes, political, social, or cultural movements and existing spread of misinformation (accidental falsehood) online. The most effective disinformation campaigns seem to be those that exacerbate or amplify misinformation campaigns.^23^ While the ongoing Russian disinformation campaign appears to be the most sophisticated and targeted example and has gained unprecedented attention, more and more literature and reports point toward other nation-states increasingly attempting to harness this tactic.

## Impact of Fake News Campaigns on Public Health

The most profound example of the consequences of fake news campaigns on public health is harmful effect of Russian disinformation campaigns and cyberattacks on US public health systems, through their contribution to the erosion of trust in traditional public health measures. The major area of focus for Russian fake news campaigns, alongside the divisive targeting of race relations and immigration, was vaccination. Between July 2014 and September 2017,^24^ Russian trolls posted online content about vaccination at a higher rate than the average user; furthermore, researchers identified a particular campaign that used the hashtag #VaccinateUS. Accounts using this hashtag in their posts were almost exclusively associated with accounts attributed to the Russian Internet Research Agency, the Russian state-linked company identified as the central disinformation producer during this period. Russia-linked “content polluter accounts,” or bots that hijack an ongoing conversation or debate for political or commercial purposes,^25^ posted memes that were later picked up and widely shared by existing antivaccine communities.^24^ Some of these tweets included “#vaccines are a parent's choice. Choice of color of a little coffin. #VaccinateUS” and “Did you know there was a secret government database of #vaccine-damaged children? #VaccinateUS.”^26^

Although Russian disinformation accounts targeted both sides of the vaccination debate,^24^ the effect of the antivaccination tweets and the normalization of the fake news economy brought new momentum and new confirmation to the antivaccination movement in the United States. In their book *A Lot of People Are Saying*, Muirhead and Rosenblum^23^ identify 3 mental processes that create the disposition to believe and understand fake news: (1) intentionality – it is much easier for people to believe that circumstances are the effect of effort than random consequence; (2) proportionality – when something significant happens, people prefer to believe that the cause of that event was similarly significant; and (3) confirmation bias – “when it comes to true enough, what matters is not evidence but repetition.” The Russian “firehose of falsehood” was categorized by a model of rapid, repetitive, and continuous communication as playing to this strategy of repetition.^27^ This model relies on the use of continual messaging on a topic from different sources, either different social media trends or different accounts on social media, to underscore a common assumptions that if you hear something from multiple sources it is more likely to be true and if you hear something multiple times you also are more disposed to accept its truthfulness. Dependent on and coupled with this rise of the fake news economy has been the delegitimization of traditional authorities and media. In 2017, two-thirds of Americans reported that they got at least some of their news from social media, either through content algorithms that provided stories that support previously expressed political or social positions or from other social media users, who typically share similar opinions.^14^ This move took online communities past the traditional concept of community-based echo chambers to distinct groups that create and experience specific realities.^28^

Although the antivaccination movement has existed since the development of vaccines, often using similar arguments as its modern counterparts such as state involvement and health concerns, its modern influence is largely traceable to a now discredited paper by ex-physician Andrew Wakefield, which falsely claimed to have identified a link between the measles, mumps, and rubella vaccine and autism. This paper fell on fertile ground at the beginning of the Web 2.0 era, with users sharing it as an alleged scientific justification for their beliefs and using it to further an already developing confirmation bias.^29^ The paper also bolstered the beliefs of communities whose antivaccination stances were not based on scientific evidence but rather religious grounds. While in the 2010s these communities remained relatively marginalized and were not present in mainstream politics, the disinformation campaign of the US elections in 2016 reversed this. The subsequent and consequential polarization of US politics, and, in particular, the polarization of the right wing, has allowed the vaccination debate to be normalized as part of right wing mainstream political system, by moving from social media to the right-wing news sites orbiting Fox News and Breitbart News. These sites, in turn, have become key sources of information for the Republican base.^14^ This normalization campaign is evidenced in the increase of antivaccination measures: in 2019 20 US states introduced bills intended to broaden the reasons for vaccine exemption.^30^

The normalization of the antivaccination movement has had significant consequences for public health and has undermined existing public health practices and created mainstream doubt about long-standing scientific consensus.^24^ The resurgence and normalization of antivaccination debates has been mirrored in the resurgence of measles cases, which jumped globally by 30% since 2016, causing WHO to declare the antivaccination movement as a top-10 threat to global health in 2018.^31,32^ In recent years, 8 US states have seen measles outbreaks, with New York reporting over 275 cases in 2019 alone.^32^ This resurgence in cases can, therefore, be directly linked to the mainstreaming of the antivaccination argument as part of an asymmetric warfare approach, legitimized and artificially amplified using the Russian network of fake media accounts during the 2016 election process. This reached global populations as political populism grew across Western Europe where similar dynamics, including a disenfranchisement of wide parts of the population and distrust in “elites” and experts, continued to drive the antivaccination movement.^33^

Similar disinformation campaigns are beginning to be identified and reported in the context of the current COVID-19 pandemic. In late February 2020, US government officials accused Russia of using thousands of accounts across a variety of social media platforms—including Facebook, Twitter, Instagram, and TikTok—to promote fake news and conspiracy theories, the most prevalent theory being that the virus is a US-created bioweapon intended to damage China economically.^34^ In mid-March, a leaked EU report identified 80 examples of Russian disinformation campaigns related to COVID-19 claiming that the virus was a biological weapon created and deployed by China, the United Kingdom, or the United States, depending, of course, on the audience. The report stated that “[p]ro-Kremlin media outlets have been prominent in spreading disinformation about the coronavirus, with the aim to aggravate the public health crisis in western countries, specifically by undermining public trust in national healthcare systems.”^35^

China similarly has deployed disinformation campaigns, targeting the European Union's response to the coronavirus pandemic by suggesting EU countries were praising Chinese aid and by suggesting that the origins of the pandemic were originally American.^36^ China's current tactics have been described as imitating the playbook laid out by Russia during the 2016 US presidential elections.^37^ Indeed, in June 2020, Twitter removed 23,750 accounts directly attributed to Chinese disinformation operations, and a further 150,000 accounts associated with amplifying the messages of these original accounts by retweeting and liking their messages. In an analysis of these tweets conducted by the Stanford Internet Observatory, researchers identified a concerted campaign running since at least as early as October 2019, which was originally intended to spread propaganda regarding the Hong Kong protests, but then switched to spreading propaganda and misinformation related to the spread of COVID-19 in 2020.^38^ Narratives spread regarding the COVID-19 pandemic were primarily focused on praising China's response, critically contrasting the responses of Taiwan and the United States to China, calling for the United States to put aside its political biases and work with China, and using the virus as an opportunity to attack the activists in Hong Kong.^38^ EU officials have accused Russia and China of using these campaigns to fuel distrust in the European Union and to exacerbate existing political tensions and issues, such as vaccination, immigration, and the targeting of minority groups.^35^ In June 2020, European officials accused China of running a “huge wave” of disinformation campaigns inside the European Union, including spreading the rumor that care workers in France were leaving their jobs and leaving residents to die.^39^

The United States has also engaged in misinformation during the current pandemic. The identification of coronavirus as “Kung Flu” by US President Trump feeds into a US narrative of blame and anti-Chinese and isolationist sentiment, reinforced by the rhetoric of the “Chinese virus” and “Wuhan virus” pushed by the current US administration.^40^ The US administration has been responsible for significant domestic misinformation and rumors, which have had a deleterious effect on the public health efforts to control the epidemic, including repeated assertions that the pandemic will go away on its own, that it is not as serious as other widespread diseases such as seasonal influenza, that the drug chloroquine can be taken preventatively, and that drinking bleach can cure COVID-19.^41^

While it is important to note that the US misinformation spread, in particular, is targeted domestically, these narratives have a significant impact on public confidence in western public health intervention, as many of these messages align with antivaccine sentiment-, antiexpert-, and antiglobalist-linked skepticism. These messages are already being picked up by the antivaccine community and disseminated further—antiscience and antivaccine supporters created several videos on TikTok blaming the Bill and Melinda Gates Foundation for the outbreak, claiming that the organization had engineered the virus in order to increase vaccination sales. These videos were viewed over 160,000 times before being taken down by the TikTok platform.^42^ Previous research into the impact of media on public perceptions of epidemics found that during an outbreak, the thematic framing of the issues by politicians and the prevalence of misinformation have had a significant impact on public actions; uncertainty related to outbreaks can impact whether individuals undertake recommended public health behaviors, with individuals less likely to follow official public health recommendations when high levels of confusion or uncertainty exist.^43^

While the Russian campaign was originally intended to divert the US elections and foster internal disruption and civil unrest in the United States, this largely political strategy has generated significant secondary effects on public health across the United States and Europe through the reinvigoration of antivaccination movements and the exacerbation of mistrust in public health responses, especially those related to COVID-19. Although these campaigns appear untargeted at public health at present, the public health effects are consequential and meet both conditions of a biological attack: (1) a negative influence on public health linked by fear, economic and political disruption, and civil unrest, and (2) the incapacitation of a target population, who are now more vulnerable to infectious diseases such as measles. The new era of biowarfare is the merging of disinformation and biowarfare, to produce the cyber bioattack. The recent resurgence of measles appears to have been a side-effect of a disinformation campaign—a targeted campaign would be an even greater public health hazard.

## Effect of Social Media Misinformation and Disinformation

The impact of disinformation and misinformation campaigns during an epidemic can be significant. We have identified the following consequences:

- The perpetuation and persistence of transmission
- Mistrust in government responses, preventing people from seeking treatment
- Direct misinformation about the epidemiological nature of the disease, preventing people from seeking treatment
- Violence against government response facilities or healthcare personnel
- Stigmatization of those infected, leading to violence or people not seeking treatment
- Exacerbation of existing political sentiment, including antigovernment or antiforeigner sentiment
- Exacerbation of existing political movements, such as antiimmigration movements.

These consequences have been experienced and associated with the circulation of misinformation during the 2 Ebola outbreaks in Africa between 2014 and 2020. In this specific context, the spread of misinformation has been identified as a key driver in the persistence of transmission in the 2019-2020 Democratic Republic of Congo (DRC) Ebola outbreak. Rumors about the public health response in this outbreak were in part perpetuated by social media.^44^ While there is no current evidence that these were concerted disinformation campaigns, misinformation was nevertheless prevalent online. These rumors centered on notions that the virus did not exist, that it was an extermination campaign by the West and domestic elites, and that it was an organ theft plot.^44,45^ These rumors were magnified by a wider context of regional conflict and mistrust of government and military forces. Social media posts blamed the United States for bringing Ebola into Africa, and US right-wing sites such as Breitbart—in an effort to motivate antiimmigration sentiment—claimed that asylum seekers were purposefully attempting to bring Ebola into the United States.^46^

Not only did mistrust of the government and international nongovernmental organizations lead to people avoiding treatment or healthcare centers, but it also drove violence against healthcare workers, preventing the provision of care and increasing the morbidity and mortality of other diseases.^47^ A study conducted among focus groups in the DRC found that 72% of respondents were dissatisfied with and mistrustful of the public health and government response, while 15% expressed that they would not comply with public health recommendations regarding isolation, quarantine at treatment centers, and safe burial in the case of illness or death in a family member.^48^ In 2019, in the DRC, Doctors Without Borders/Médecins Sans Frontières recorded 300 attacks against healthcare workers, including an arson attack against and Ebola treatment center in Katwa.^49^

Similar misinformation circulated during the 2014-2016 Ebola outbreak in West Africa—largely in Guinea, Liberia, and Sierra Leone—both in these countries and internationally. A study found that following the diagnosis of the first Ebola case in the United States, Twitter mentions of Ebola leapt from 100 per minute to more than 6,000 per minute. Many of these posts contained misinformation relating to viral transmission dynamics as well as politicized statements against immigrants bringing Ebola into the country or reports of “the infected” running wild in American cities.^50^ In Guinea, Liberia, and Sierra Leone, a study found that the most common misinformation spread on social media was the belief that Ebola could be cured by either blood transfusion or a drink made with the ewedu plant.^51^ The prevalence of misinformation in the 2014-2016 West Africa Ebola outbreak was tracked and combatted by the DeySay app—“Dey Say” being the Liberian-English term for how people speak about rumors.^52^ The mobile app allowed healthcare workers and individuals to text the rumors that they had heard circulating to a central coordination base, which fact-checked the rumors using credible scientific and governmental sources, then produced weekly reports for media and nongovernmental organizations to share in response to the rumors. Not only did the application demonstrate to workers on the ground that the prevalence of rumors and misinformation was responsible for the crisis as much as the failure of the healthcare system, but it also allowed healthcare workers to track areas prone to particular rumors and pockets of resistance and to combat specific rumors.^52^

Overall, the effect of Ebola misinformation has been to drive social unrest through the erosion of trust in public health and to worsen the health status of the population as a consequence of negative changes in health-seeking behavior. If misinformation was able to divert public health responses and result in increased violence against healthcare workers and sustained transmission of the virus, we should anticipate and consider the potential harm that might be produced by targeted disinformation campaigns in epidemic or pandemic settings.

## The Delegitimization of Science

In a context of delegitimization of expertise by social media accounts and a sustained assault on science by populist politicians, disinformation campaigns are likely already disrupting global efforts to fight the epidemic by eroding confidence in the public health response.^34^ In a recent survey, only half of Americans state that they would get a COVID-19 vaccine if one was available.^53^ This delegitimization of public health institutions and governments has been exacerbated by disinformation and misinformation on social media, which spread rumors about the origins of outbreaks, rumors of covert aims of government bodies, and false epidemiology. As a result, public health institutions now have to consider the implications of their advice or instructions being ignored. The consequences of this for both public health institutions and the pandemic response are vast: people may choose not to follow or believe public health guidelines, instructions, or evidence from public health institutions. Previous research has suggested that the media often frames health risk messages in ways opposite to the original messaging by public health bodies, which interferes with and may change the public's perception of risk. Media framing is often impacted by ideological leaning and bias of previous coverage, which can misconstrue or deconstruct scientific evidence.^43^ In response to the use of quarantine as a public health measure to decrease coronavirus transmission across the United States, groups of people protested, claiming that the virus transmission was fraudulent, lesser than claimed, or not as important as the economic damage of closing the economy.^54^

## Information and Biowarfare

The use of biological weapons to disrupt national security can refer to either the introduction of an existing disease or engineered pathogen deployed against a population or state to cause insecurity. Such matters are governed by the 1925 Geneva Protocol^55^ and the 1972 Biological Weapons Convention^56^—formerly known as the Convention on the Prohibition of the Development, Production, and Stockpiling or Bacteriological (Biological) and Toxin Weapons and on their Destruction. Despite the use of biological weapons in military campaigns throughout history, their use has not been greatly expanded the 20th century for 3 reasons: the existence of strong normative and cultural barriers to their use; difficulties storing and deploying them, which prevents their assimilation into conventional military arsenal; and the political and strategic fear of retaliation, escalation, and international reaction.^1^

The effects of disinformation campaigns on public health can produce consequences potentially comparable to biological warfare and terrorism: to weaken and undermine an opponent or to cause disruption and panic within a population. The legitimization of antivaccination campaigns has contributed to the rise in measles cases, and the erosion of trust in medicine and public health measures has led to a dramatic impact on the ability to deliver effective medical care and has exacerbates existing political and social divisions. In the case of COVID-19, disinformation campaigns have contributed to growing panic and disruption, weakening public health and epidemic control measures. Importantly, the main aims of biological warfare—the causation of civil unrest, economic, and political disruption and the weakening of a target population—have been achieved by disinformation campaigns. Crucially, these campaigns have achieved this without triggering the identified conditions precluding them from wider use.

The use of disinformation campaigns has been normalized within public consciousness; not only did Russia use disinformation to target the US elections, but there is evidence of attempted interference in elections in France, Germany, and the Netherlands, as well as the United Kingdom's Brexit referendum.^57^ The normalization of misinformation and disinformation has not yet been accompanied by the establishment of normative or adapted regulatory systems. At present, such campaigns can easily be assimilated into military use, with the majority of militaries globally seeking to advance their cyber capabilities. Furthermore, there has been, as yet, little concerted international action concerning Russian cyber-bio operations. Legislative actions against the use of so-called deep fakes and disinformation campaigns are growing domestically, with impacts on national security being slowly considered; however, when international norms will be established remains relatively unclear.^58^ The US intelligence communities conducted investigations into the Russian disinformation campaign in 2016, resulting in the indictment of several individuals and the Internet Research Agency, the government-linked organization responsible for the majority of disinformation campaigns operating prior to the 2016 election. Measures to stop such campaigns have been undermined by the refusal of the US president to believe or support these investigations. The lack of evidence or the refusal to believe or act on existing evidence has contributed to inactivity on the part of other governments as well. The French “Macron Leaks” were never formally attributed to Russia and the UK government has refused to release the results of the investigation into Russian influence in Brexit. Although all governments claim to have addressed the matter with the Russian President Vladimir Putin, no further formal indictments or actions have been made, as international legislation remains largely ill-equipped to combat these measures.^59^

## Conclusion

Disinformation campaigns, including those identified in this article, have targeted the erosion and undermining of public trust in political and public health processes. We suggest that the consequential nature of these campaigns requires a necessary shift in biowarfare analysis and that the growing domain of misinformation and disinformation should be considered in strategic nation-state practices as a form of biological threat. It is possible that the public health consequences of such campaigns are targeted outcomes of evolving geopolitical strategy and that arguments of unintended consequences increasingly lack credibility. We suggest that by using disinformation campaigns, nation-states can produce the consequences of biological terrorism and warfare without the technical and regulatory ramifications of their use. The identified Russian-linked campaigns represent the advent of a fifth biowarfare era: a combined cyber biowarfare able to replicate the effects of a biological agent while remaining outside of existing normative frameworks. It is possible to conceive of the catastrophic impact of a disinformation campaign directly targeted at public health that—drawing on fake news and misinformation—could divert the course of an epidemic by preventing people from accessing treatment, increasing civil conflict (eg, by blaming a particular population for the creation of an alleged bioweapon), and provoking attacks on health workers. The lack of international norms relating to the regulation of these campaigns has resulted in hostile actors remaining largely free of concrete international reprimand. The increase in measles outbreaks, the 2014-2016 West Africa Ebola outbreak, and the current COVID-19 pandemic continue to demonstrate how dangerous “infodemics” are to public health and state stability. Improvements in cyber regulations for health and security are crucial to the sustainability and coherence of current frameworks targeting the interface of natural and engineered biological threats.
